# Chemical and Energetic Characterization of the Wood of *Prosopis laevigata*: Chemical and Thermogravimetric Methods

**DOI:** 10.3390/molecules29112587

**Published:** 2024-05-31

**Authors:** Luis Fernando Pintor-Ibarra, José Juan Alvarado-Flores, José Guadalupe Rutiaga-Quiñones, Jorge Víctor Alcaraz-Vera, María Liliana Ávalos-Rodríguez, Oswaldo Moreno-Anguiano

**Affiliations:** 1 Facultad de Ingeniería en Tecnología de la Madera, Universidad Michoacana de San Nicolás de Hidalgo, Edif. D. Cd. Universitaria, Santiago Tapia No. 403, Centro, Morelia 58000, Mexico; jose.rutiaga@umich.mx (J.G.R.-Q.); 0234033j@umich.mx (O.M.-A.); 2 Instituto de Investigaciones Económicas y Empresariales, Universidad Michoacana de San Nicolás de Hidalgo, Edif. D. Cd. Universitaria, Santiago Tapia No. 403, Centro, Morelia 58000, Mexico; jorge.alcaraz@umich.mx; 3 Centro de Investigaciones en Geografía Ambiental, Universidad Nacional Autónoma de México, Antigua Carretera a Pátzcuaro No. 8701, Col. Ex Hacienda de San José de la Huerta, Morelia 58190, Mexico; lic.ambientalista@gmail.com

**Keywords:** TGA-DTG, biomass, proximate analysis, calorific power, biofuels

## Abstract

Diverse methodologies exist to determine the chemical composition, proximate analysis, and calorific value of biomass. Researchers select and apply a specific methodology according to the lignocellulosic material they study and the budgetary resources available. In this project, we determined the primary chemical constitution and proximate analysis of *Prosopis laevigata* (Humb. & Bonpl.) Jonhst wood using a traditional chemical method and a novel procedure based on the deconvolution of the DTG signal produced by TGA. The highest calorific value was verified using a calorimetric pump based on mathematical models. We also conducted elemental analysis and a microanalysis of ash, and applied Fourier transform infrared spectroscopic analysis (FT-IR). The means of the results obtained by the chemical method and TGA-DTG, respectively, were: hemicelluloses 7.36%–(8.72%), cellulose 48.28%–(46.08%), lignin 30.57%–(32.44%), extractables 13.53%–(12.72%), moisture 2.03%–(4.96%), ash 1.77%–(1.90%), volatile matter 75.16%–(74.14%), and fixed carbon 23.05%–(18.93%). The procedure with the calorimetric pump generated a calorific value above 20.16 MJ/kg. The range generated by the various models was 18.23–21.07 MJ/kg. The results of the elemental analysis were: carbon 46.4%, hydrogen 6.79%, oxygen 46.43%, nitrogen 0.3%, and sulfur 0.5%. The microanalysis of ash identified 18 elements. The most abundant ones were potassium ˃ calcium ˃ sodium. Based on the infrared spectrum (FT-IR) of *Prosopis laevigata* wood, we detected the following functional groups: OH, C-H, C=O, CH_2,_ CH_3_, C-O-C, C-OH, and C4-OH. Our conclusion is that the TGA-DTG method made it possible to obtain results in less time with no need for the numerous reagents that chemical procedures require. The calorific value of *P. laevigata* wood is higher than the standards. Finally, according to our results, proximate analysis provides the best model for calculating calorific value.

## 1. Introduction

Worldwide, 44 species of the genus *Prosopis* have been registered. The predominant species in Mexico are *P. laevigata* and *P. glandulosa*. In this project, we studied the wood of *P. laevigata*, a species of the Fabaceae family that has a broad, natural distribution in arid and semiarid zones of the country. Estimates indicate that 50% of Mexico’s national territory has these types of climates, concentrated in the states of Guanajuato, Hidalgo, Michoacán, Nuevo León, Oaxaca, Puebla, San Luis Potosí, and Veracruz [1,2]. *P. laevigata* is a species of great ecological and economic importance. Its wood is commercialized mainly in the manufacture of rustic furniture, artisanal crafts, flooring, railroad ties, moldings, doors, and windows, as well as charcoal production. The bark is used in tanning, the exudate known as gum arabic is used in cosmetic products, printing, and as a glue for paper, while the leaves and fruits have medicinal applications as antimicrobials and are utilized in cattle feed [3,4]. While information on the uses, diversity, and distribution of this wood is abundant, there are few studies of its chemical constituents and energy properties [5,6,7]. Given the heterogeneous nature of wood, it is especially important to determine the chemical and energy properties of its lignocellulosic biomass as a possible energy source. A renewable raw material, wood is neutral in terms of emissions and ensures a sustainable supply because the CO_2_ it produces is equivalent to the amount of energy retained in the plants [8,9]. Today, bioenergy is the main source of renewable energy, representing around 10% of global energy consumption and 77% of all forms of renewable energy; that is, biofuels and hydroelectric, solar, eolic, and geothermic energy [10]. Determining the chemical and energy properties of this biomass can be achieved using various techniques that evaluate its principal chemical components, but proximate analysis of lignocellulosic materials using routine gravimetric procedures require considerable care and time [9,11]. Other methods include thermogravimetric analysis (TGA) and the deconvolution of the first derivative (DTG), which generate precise quantifications in less time, and can determine the primary constituents of biomass and proximate analysis [12,13]. Few researchers, however, have access to DTG because many laboratories lack the budgetary resources to acquire the equipment used to perform TGA. For this reason, they continue to use classic gravimetric methods for the chemical analysis of biomasses [9,14]. The objective of the present study is to contribute to the development of new methodologies that will improve our knowledge of the chemical and energy properties of *P. laevigata* wood.

## 2. Results and Discussion

### 2.1. Primary Constitution of the Wood: Chemical and TGA-DTG Methods

Diverse methodologies are available to determine the primary composition of biomass, but it is well known that each one has certain strengths and weaknesses [9,11]. This study found that statistically significant differences exist (*p* ≤ 0.05) between the chemical and thermogravimetric (TGA-DTG) methods for the parameters of the primary constituents—hemicelluloses and lignin– and in a proximate analysis of moisture, volatile matter, and fixed carbon. In contrast, there were no significant differences for cellulose, extractables, and ash (Table 1). The differences encountered are related to the procedure used, since the chemical method makes its determinations individually, while thermogravimetry (TGA-DTG) obtains results through pyrolysis in a controlled system using mathematical calculations, such as multiple linear regression, where the fractions of the main chemical components can be optimized [15]. Independent thermal degradation by pyrolysis of the three principal chemical components of wood (cellulose, hemicelluloses, and lignin) can accurately predict the parameters of deconvolution of the DTG [13,16].

Our review of other chemical studies of the duramen of *P. laevigata* wood using chemical methods found that published results vary. Carrillo et al. [5], for example, reported the following percentages: hemicelluloses 16%, cellulose 45.7%, lignin 31.4%, and extractables 16%. In contrast, based on a fiber analysis by the Van Soest method [7] published these yields: hemicelluloses 14.09%, cellulose 45.35%, lignin 22.14%, extractables 16.72%, and ash 1.70%. The results of our work are only comparable with respect to cellulose, lignin, and ash, according to these references (Table 1). It is important to emphasize that the relevant literature indicates that the chemical constituents of wood can vary by the material studied (duramen, sapwood, bark), the age of the tree, growth conditions, geographic location, altitude, climatic factors, the chemical solvents utilized, and experimental methodologies, among other factors [17,18].

**Figure 1:**
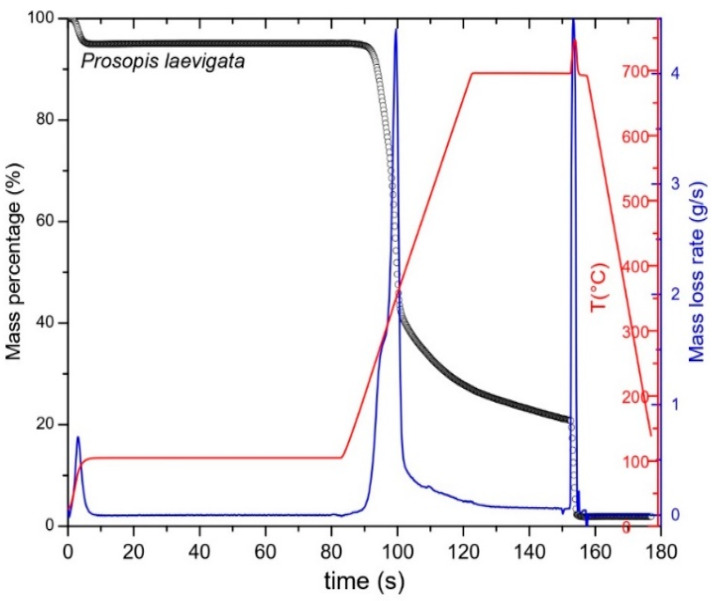
TGA and DTG curves of *P. laevigata* wood.

Through proximate analysis, one can obtain an immediate profile of the quality of solid biofuels, so this is an important approach for commercialization and the design of combustion equipment [19], two parameters that affect the quality of biofuels. For example, high percentages of moisture and ash reduce the calorific value and efficiency of biomasses during combustion [20,21], while high percentages of volatile matter increase yields during the conversion of biomass into biodiesel by pyrolysis and favor the production of biogas and the calorific value in densified biofuels [22,23]. In this regard, high fixed carbon content improves the calorific value of densified biofuels and charcoal [24,25]. Our work did not find any statistically significant differences for ash content (Table 1). The statistically significant differences determined for the other parameters tested in the proximate analysis may be related to the procedures used in distinct methodologies; for example, the fact that in TGA pyrolysis is determined using a sample in a controlled system (Figure 1). However, they may also be attributable to the calculation method applied. In gravimetric methods, these are performed on the basis of biomass that is free of moisture using, for instance, the expression: % fixed carbon = [100 − (% ash + % volatile matter)], where adjusting for the moisture in the ash and volatile matter is performed previously. Meanwhile, the TGA method for calculating volatile matter uses the expression: % volatile matter = [100 − (% moisture + % ash + % fixed carbon)]. Based on these expressions, it is clear that the adaptation of the % of moisture influences results.

Because *P. laevigata* wood has an especially high amount of fixed carbon, it is a solid biofuel of good quality. The proximate analysis of the duramen of *P. laevigata* in a study using gravimetric methods obtained the following percentages: ash 1.70%, volatile matter 78.46%, and fixed carbon 19.83% [7]; while in their work, Martínez-Pérez et al. [6] reported 1.24% of ash in the duramen of this species. In comparative terms, only the percentage of ash obtained in these two reports is comparable to the references consulted (Table 1). In general, the results obtained with the two methods applied in our work are within the ranges reported for woods of latifoliate species: ash 0.49–4.51%, volatile matter 76.60–94.70%, and fixed carbon 9.32–21.27% [26,27].

### 2.2. Thermal Decomposition Process (TGA) and Deconvoluted (DTG)

In the first stage of the pyrolysis process of *P. laevigata* wood, conducted in the temperature range of 25–100 °C for a period of 80 min, a loss of moisture from the biomass occurred (Figure 1). Some studies have reported that this temperature causes the volatilization of the organic fraction of low molecular weight biomass, including some extractable substances [28]. 

**Figure 2:**
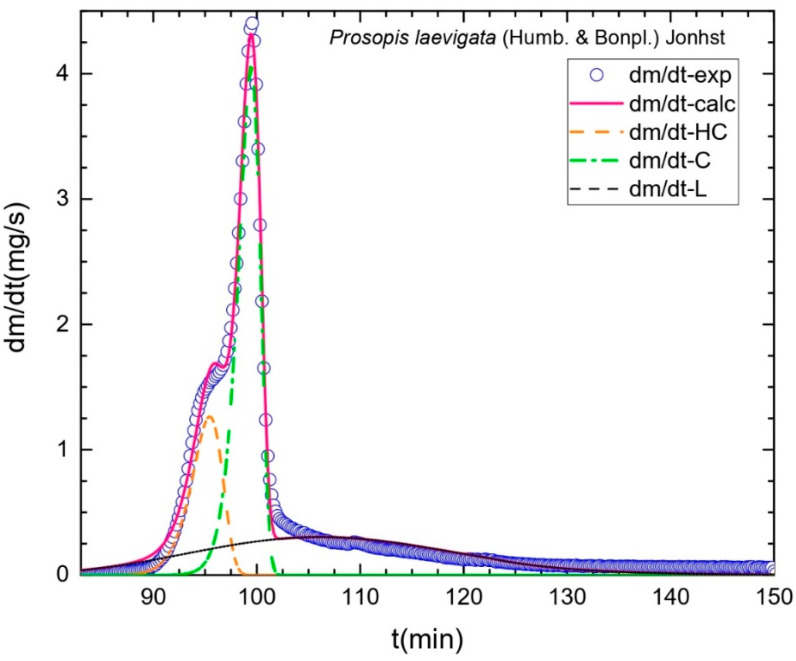
Deconvoluted DTG curve of *P. laevigata* wood.

In the second stage, when the temperature was increased from 100 to 700 °C for 30 min, we obtained the calculation of the fixed carbon content from the proximate analysis. This stage included the thermal decomposition of the hemicelluloses and cellulose. It is estimated that the loss of mass of these polymers occurs in a temperature range of 200–675 °C [29,30]. Another characteristic of this stage is a greater loss of the wood fraction. As Figure 1 shows, DTG indicates the degradation velocity of the mass (in blue). It is well known that wood is made up mainly of these polysaccharides, with estimated fractions as high as 70% [18,31]. Table 1 shows the percentages of these chemical constituents. Figure 2 presents the deconvolution of the DTG, where the orange color represents the primary component with lower thermal stability due to the presence of acetyl groups that correspond to the hemicelluloses. Other researchers have reported this tendency [12,15] in other lignocellulosic materials. The curve of greater size—in green—represents cellulose. This second polymer presents higher thermal resistance than the hemicelluloses due to its crystalline structure [32,33].

This illustration includes a curve—in black—that corresponds to lignin, which began a reaction at low temperatures that persisted across a broad temperature range. This phenomenon is associated with the diverse functional groups in its structure, which vary in terms of thermal stability [34]. Reports in the literature include antecedents of this phenomenon of thermal degradation in some latifoliate species [12,32]. Figure 2 also shows the experimental graph—in blue—with calculations in pink. The similarity between them is evident, leading to the conclusion that they are correct. In the third stage (Figure 1), a peak was registered at an approximate time of 155 min. This corresponded to the influx of oxygen that was applied at 700 °C to eliminate all organic matter. Finally, the last fraction, consisting of the remaining residue, corresponds to the inorganic fraction made up of ash, which was obtained at the end of the cooling process from 700–25 °C in a time of 20 min.

### 2.3. Higher Heating Value (HHV)

The calorific value is the amount of energy per unit of mass or volume that is released in a complete combustion [35]. It is the most important parameter for characterizing materials used as fuels. The calorific value of wood depends on its chemical components. Some authors mention that lignin favors the calorific value of solid biofuels [36,37], though other studies have concluded that extractables increase calorific value, especially in species of *Pinus* spp. [38,39], while others report this same effect [40]. Our study did not identify any statistically significant differences (Tukey, *p* ≤ 0.05) in the calorific value higher than—using a calorimetric pump—between the predictive mathematical models based on chemical composition and the proximate analysis (Table 2). We did, however, find that the model based on elemental analysis generated a lower value and presented statistically significant differences (*p* ≤ 0.05) with respect to both the standard methodology and the other predictive models. These results are consistent with those reported in published studies that utilized the same models [26]. 

According to the results of the present study, the best model for predicting calorific value is proximate analysis, since there were no statistically significant differences between this model and the procedure that involved the use of the calorimetric pump. Moreover, this can be calculated by immediate analyses that do not require chemical reagents or large investments in expensive equipment. This concurs with reports by Rodríguez-Romero et al. [14]. Martínez-Pérez et al. [6], meanwhile, reported a lower calorific value of 18.31 MJ/kg in *P. laevigata* wood based on the approach using a calorimetric pump. They also obtained a larger interval (18.4–19.2 MJ/kg) than the one registered for latifoliate woods in UNE-EN ISO 17225-1 [41]. It is important to emphasize that the results for the caloric value obtained herein with the methodologies utilized are within the interval of 18.13–21.7 MJ/kg estimated for various species of latifoliate woods in Mexico [27,42]. According to international parameters (Table 3), the calorific value higher than of *P. laevigata* wood is above the quality parameter. 

**Table 2:** Calculation of the higher calorific value (MJ/kg) of *P. laevigata* wood.

HHV using calorimetric pump [43]	20.16 (±0.25) a
HHV = 17.9017 + 0.07444 (L) + 0.0661 (E) [44]	21.07 (±0.06) a
HHV = 354.3 (FC) + 170.8 (VM) [45]	21.06 (±0.07) a
HHV = 0.335 (C) +1.423 (H) − 0.154 (O) − 0.145 (N) [46]	18.23 (±0.92) b

Different letters in the column indicate that there is a significant Tukey statistical difference (*p* ≤ 0.05). Where: L = lignin, E = Extractives, FC = Fixed carbon, VM = Volatile material, C = carbono, H = Hydrogen, O = Oxygen, N = Nitrogen.

**Table 3:** Energetic properties, elemental analysis and microanalysis of ashes from *P. laevigata*.

Parameter	Magnitude	Calculated Value	Technical Parameters Limit [47]	Typical Variation for Hardwood Woods [41]
*Energetic properties*				
Moisture	%	7.36 (±0.15)	≤10	NS
Ashes	%	1.77 (±0.07)	≤1.5	0.2–1.0
Calorific value	MJ/kg	20.1 (±0.25)	16.3–19	18.4–19.2
*Elementary analysis*				
Carbon	%	46.4 (±0.42)	-	48–52
Hydrogen	%	6.79 (±0.15)	-	5.9–6.5
Oxygen	%	46.43 (±0.38)	-	41–45
Nitrogen	%	0.3 (±0.12)	≤0.5	˂ 0.1–0.5
Sulfur	%	0.05 (±0.01)	˂0.03	˂0.01–0.05
*Ash microanalysis (parts per million, ppm)*				
K	mg/kg	182,962.57	-	500–1500
Ca	mg/kg	122,616.47	-	800–20000
Na	mg/kg	11,042.69	-	10–200
Sr	mg/kg	3174.39	-	NS
S	mg/kg	1265.70	-	NS
Mg	mg/kg	972.01	-	100–400
P	mg/kg	649.19	-	50–200
Ba	mg/kg	106.76	-	NS
B	mg/kg	84.35	-	NS
Si	mg/kg	69.56	-	100–200
Fe	mg/kg	53.47	-	10–100
Cu	mg/kg	44.28	-	0.5–10
Al	mg/kg	35.98	-	˂10–50
Li	mg/kg	32.40	-	NS
Mn	mg/kg	23.72	-	83
Zn	mg/kg	16.37	≤10.0	5–100
Ni	mg/kg	7.70	≤10.0	˂0.1–10
Cr	mg/kg	4.48	≤10.0	0.2–10

NS = not specified.

### 2.4. Elemental Analysis 

The elemental analysis of biomass is another important parameter since it can define the exact atomic elements that make up wood; namely, carbon, hydrogen, nitrogen, and sulfur. Criteria for the quality of biofuels registered in the literature include high percentages of C and O in biomass, as this favors calorific value [48,49]. This affirmation must be qualified; however, because Ali et al. [50] reported that lignocellulosic materials with high O content can corrode combustion equipment. Hydrogen, in contrast, has been found to contribute positively to calorific value, though its proportion in biomass is lower than that of C [48]. Studies also estimate that high amounts of N and S in biomass are the main causes of environmental impacts that can damage human health and lead to the formation of incrustations in combustion equipment [51]. Table 3 presents the results of our elemental analysis of *P. laevigata* wood, which determined a low proportion of C, while H and O were above the ranges reported in the literature for latifoliate woods: respectively, % C: 49.13–50.63, % H: 5.96–6.21, and % O: 43.62–44.49, compared to Rutiaga-Quiñones et al. [43]. Based on norm UNE-EN14961-1 [47] for solid biofuels, N complies with this parameter, but S is slightly above the limit and is comparable to the range estimated for woods of latifoliate species (Table 3). 

### 2.5. Microanalysis of Ash 

This analysis was performed to identify the inorganic fraction of the biomass, which consists of the minerals present in the soils where the trees grew. Although these exist in low proportions in wood, they can have negative effects on combustion. For example, Ca, K, P, and Mg can produce slag, corrosion, and fine particle emissions, and form incrustations in combustion equipment [52,53]. Na, Fe, and Si can cause problems like ash fusion, incrustations, and corrosion [54]. We identified 18 elements in our samples (Table 3). The most abundant ones were K ˃ Ca ˃ Na. Published studies have reported that Ca, K, P, and Mg are the most abundant chemical elements in wood ash [26,39]. This is consistent with our identifications. Sjöström [17] mentions that B, Cu, Mn, Si, and Zn can be found in lower proportions, and, in effect, we detected these elements. In relation to the elemental ranges of solid biofuels listed in norm UNE-EN 14961-1 [47], *P. laevigata* wood exceeds the ≤10.0 mg/kg of Zn. In contrast, Ni and Cr concentrations were below reported levels (Table 3). Other microanalyses of the wood ash of *P. laevigata* have also detected the presence of Ca, K, Mg, P, Si, and Al [6]. 

### 2.6. Fourier Transform Infrared Spectroscopy (FT-IR) 

**Figure 3:**
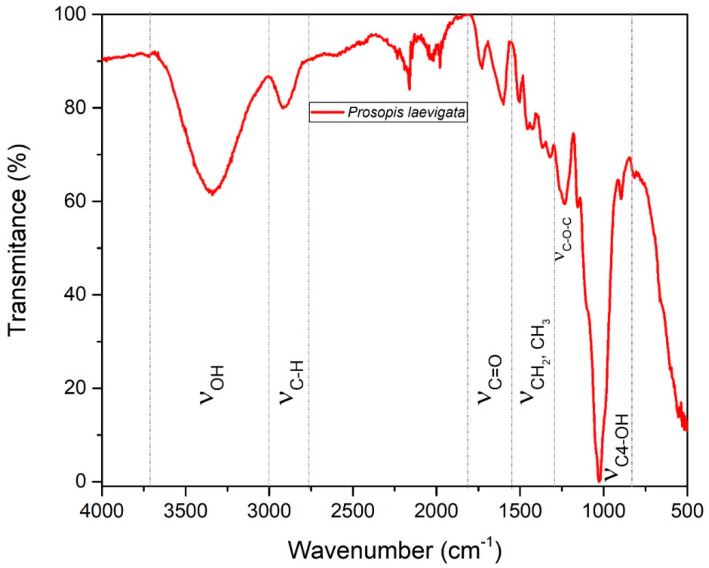
FT-IR infrared spectrum of *P. laevigata* wood.

Figure 3 shows the infrared spectrum (FT-IR) of *P. laevigata* wood. We identified the OH group in the 3700–3000 cm^−1^ region. This functional group has also been reported for *P. juliflora* wood from our study area [55]. Published reports suggest that this functional group corresponds to the water molecules that constitute the biomass. However, it is important to note that significant broadening of this band indicates the presence of numerous hydrogen bonds. The existence of adsorbed water molecules or crystallization may be considered less important. This has been reported for other latifoliate woods at 3423 cm^−1^. These stretching vibrations are related to the cellulose and hemicellulose polymers [56] in the absorption band of the 3000–2750 cm^−1^ region. We also identified the functional group CH and, as with other species of this genus, there was an absorption band in the 2980–2800 cm^−1^ region in *P. juliflora* wood [55]. According to the literature, absorption bands in this region are related to the CH group present in the structural components of wood (cellulose, hemicelluloses, lignin). The CH functional group has been found in other studies of latifoliate species, in the region of 2933 cm^−1^ [57,58]. The peak found between wave numbers 1800–1500 cm^−1^ is related to the C=O stretching vibrations of carboxylic acid and/or carbonyl (aldehydes, ketones, and esters) [59]. This result is comparable to other studies of latifoliate woods that report the presence of this functional group between 1740 and 1730 cm^−1^. It is important to note that this functional group has been assigned to the hemicelluloses [58,60]. 

Turning to the carbonyl functional group, it was identified at an absorption of 1695 cm^−1^ in extracts isolated with acetone, and in aromatic substances between 1615 and 1520 cm^−1^ in extractable substances obtained with methanol and water in *P. kuntzei* wood. Other published reports affirm that these low molecular weight organic compounds are often present in derivatives of gum arabic, mainly in species of the genus *Prosopis* [61]. The position of the absorption bands between 1510 and 1260 cm^−1^ is associated with the functional groups CH_2_ and CH_3_, which correspond to the FT-IR spectrum of this wood. The CH_2_ group has also been identified in *P. juliflora* wood [55]. These functional groups are associated with cellulose and lignin [58,62]. Other studies have detected them in latifoliate species in the region of 1464–1375 cm^−1^ [59,63]. Finally, the functional groups C-O-C, C-OH, and C4-OH were identified in vibrations at 1234, 1160, and 1028 cm^−1^, respectively, and β-glucopyranose was found in the cellulose [64]. Similar values have been reported for other latifoliate species, including beech [65]. 

## 3. Materials and Methods

### 3.1. Collection and Preparation of the Study Material

Samples of *P. laevigata* wood were collected in the Lake Cuitzeo Basin in the State of Michoacán, Mexico, between parallels 19°58′51” N 101°7′15″ O and 19°58′21” N 101°7′28″ O. They were taken from three individual trees of this species in 10-cm slices, cut at a height of 1.30 m (diameter at the height of the chest). The wood was separated from the bark, splintered manually, and left to dry in the shade until it reached a moisture equilibrium (approximately 12%). After drying, the splinters were ground in a conventional grinder, and then sieved in a RO-TAP device (Model RX-29, W.S. Tyler, Mentor, OH, USA) using standard meshes, as follows [66]: numbers 20, 40, and 60, and the #40 flour mesh (425 µm), in accordance with norm T 264 cm-97 [67]. The higher heating value (HHV) was utilized for the chemical, thermogravimetric (TGA), proximate analysis, elemental, and calorific analyses. 

### 3.2. Primary Constitution and Proximate Analysis by Chemical Methods

#### 3.2.1. Primary Analysis by the Chemical Method

Chemical characterization was conducted in an Ankom Model A200 fiber analyzer (ANKOM Technology, Macedon, NY, USA) following the methodology in Van Soest et al. [68]. Gravimetric calculations were performed to obtain the percentages of neutral detergent fiber (NDF), acid detergent fiber (ADF), insoluble lignin (IL), and ash, based on Equations (1)–(4), as reported by Torres et al. [69].

(1) %Extractives=100−%NDF

(2) %Hemicelluloses=%NDF−%ADF

(3) %Cellulose=%ADF−%IL

(4) %Lignin=%IL−%Ash

#### 3.2.2. Proximate Analysis by Gravimetric Methods

The percentage of moisture was determined using a thermobalance (Benetech Inc., model Gm640, Aurora, IL, USA), according to norm ASTM D4442-20 [70]. The percentage of ash was measured according to norm ASTM D1102-84 [71], while volatile matter content was verified based on norm ASTM E872-82 [72]. Fixed carbon was calculated as the following difference: % fixed carbon = [100 − (% ash + % volatile matter)], based on calculations reported previously by Ngangyo-Heya et al. [73]. 

### 3.3. Thermogravimetric (TGA) and Differential (DTG) Analyses

For the purposes of this study, a rapid methodology that integrated TGA-DTG analysis was applied using a PerkinElmer STA 6000 thermogravimetric analyzer (Pyris™ software, Version 11, PerkinElmer, Waltham, MA, USA), based on the parameters of the ASTM D5142-90 standard [74]. This analyzer has an alumina crucible that was filled with an amount of 30 ± 10 mg. The sample was distributed uniformly inside the crucible. The analysis was carried out in an inert atmosphere that required a flow of nitrogen gas (N_2_) that was 99.99% pure. Before each experiment, the equipment was purged with N_2_ for 15 min to eliminate, insofar as possible, the air inside the chamber where pyrolysis was conducted. 

#### 3.3.1. Proximate Analysis by TGA 

The heating ramp was programmed in four stages. Stage (1): initial heating from 25 to 105 °C. This temperature was maintained for 80 min. The heating velocity was 30 °C/min, in a nitrogen atmosphere at a flow rate of 30 mL/min. Stage (2): temperature was increased to 700 °C at a rate of 15 °C/min, then maintained for 30 min under the same nitrogen flow. Stage (3): oxygen (O_2_) was introduced at a flow rate of 30 mL/min. The temperature was kept at 700 °C for 5 min. Stage (4): a switch was made back to nitrogen. Cooling from 700 to 25 °C was performed at a velocity of 30 °C/min. In this final stage, the nitrogen flow was reduced to 15 mL/min.

#### 3.3.2. Primary Constitution: Deconvolution of the DTG

Based on the algorithm developed and data from the TGA analysis, the fractions of the structural components of the wood (cellulose, hemicelluloses, lignin) were calculated utilizing the deconvolution of the curve of the TGA derivative that corresponded to the DTG analysis. The algorithm allowed us to determine the fractions of the structural components as a function of the loss of mass in the sample. 

The graphs of the curves were created based on the specific algorithm written in the SciLab platform, which utilizes the ODE (ordinary differential equations) subroutine based on the Adams method (for non-rigid ODE problems). This approach integrates ordinary differential equations to describe the loss of weight associated with the degradation of hemicellulose, cellulose, and lignin, and the *Fminsearch* subroutine (based on Nelder and Mead’s algorithm) to optimize the kinetic parameters and achieve the best adjustment of the experimental results [12]. Extractable substances were calculated as the following difference: % extractables = 100 − (% cellulose + % hemicelluloses + % lignin + % ash). 

### 3.4. Calorific Value Higher Than (HHV) 

The calorific value was determined in a calorimetric pump (LECO AC 600, LECCO Corporation, St. Joseph, MI, USA) following norm UNE-EN ISO 18125 [43]. The calorimeter was calibrated with benzoic acid before initiating the analysis. HHV was calculated using mathematical models according to the chemical composition (lignin and extractables), following White [44] and incorporating the results of the proximate analysis as a function of the fixed carbon and volatile matter. This procedure followed Cordero et al. [45] and employed the mathematical model that resulted from the elemental analysis [46].

### 3.5. Elemental Analysis 

Carbon, hydrogen, nitrogen, and sulfur content were determined in a Perkin-Elmer, model 2400 CHNS-Or apparatus, using the modified Dumas method [75]. Sulfur content was calculated by the turbidimetric method with gum arabic. Oxygen content was calculated by difference, based on the reports by Ghetti et al. [76]. 

### 3.6. Microanalysis of Ash 

Identification of the inorganic elements in the ash was conducted by means of inductively coupled plasma atomic emission spectroscopy (ICP-AES) (VARIAN 730-ES, Varian Inc., (Agilent), Mulgrave, Australia), under the operating conditions reported by Arcibar-Orozco et al. [77]. 

### 3.7. Fourier Transform Infrared Spectroscopy (FT-IR)

The functional groups of *P. laevigata* wood were obtained by FT-IR in a Perkin Elmer ATR model 400 spectrometer. The spectra were determined in 16 scans per sample in a range of 4000–500 cm^−1^ at a resolution of 4 cm^−1^, according to the operating conditions reported by our research team in previous studies [15].

### 3.8. Statistical Analyses 

The analyses of the primary constituents and the proximate analysis by the chemical method and thermogravimetry (TGA-DTG) were performed in triplicate. A student’s t-test was used to compare the two treatments for each variable of the basic chemical composition and proximate analysis. For calorific value, an analysis of variance (ANOVA) (*p* ≤ 0.05) was applied with a comparison of means using the Tukey test. All statistical analyses were performed with the R Studio program [78]. For the elemental analysis, we report the means and their standard deviation. The microanalysis of ash and spectroscopy (FT-IR) was carried out only once.

## 4. Conclusions

The novel TGA-DTG thermogravimetric method tested in this study can obtain results for the principal chemical components of wood and for proximate analysis in less time, once the algorithm has been obtained, with no need for numerous chemical reagents compared to traditional chemical procedures. We found statistically significant differences (*p* ≤ 0.05) between these two methods in some parameters, but both approaches yielded results comparable to reports in the literature. The calorific value of *P. laevigata* wood is higher than international standards for solid biofuels. According to our results, the best model for calculating calorific value is the one obtained through proximate analysis, as this showed no statistically significant differences (*p* ≤ 0.05) with studies that used a calorimetric pump. This method has two advantages: (i) it generates results immediately because no chemical reagents are required, and (ii) it does not require costly equipment. Regarding the elemental analysis, *P. laevigata* wood complies with the limit on nitrogen content for commercialization, while sulfur content was only slightly above the corresponding specification. The microanalysis of ash identified 18 elements, the most abundant ones being potassium ˃ calcium ˃ sodium. According to technical parameters, nickel and chrome concentrations comply with international allowances. Finally, the FT-IR analysis detected eight functional groups: OH, C-H, C=O, CH_2,_ CH_3_, C-O-C, C-OH, and C4-OH. 

## Figures and Tables

**Table 1:** Chemical composition and proximate analysis of *P. laevigata* wood by two methods.

Parameter	Chemical Analysis *		TGA-DTG Analysis *	
	Average (%)	SD (±)	Average (%)	SD (±)
Hemicelluloses	7.36	0.15	8.74	0.22
Cellulose	48.28	1.0	46.08	0.25
Lignin	30.57	0.74	32.44	0.40
Extractives	13.53	0.12	12.72	0.73
Moisture	2.03	0.14	4.96	0.11
Ashes	1.77	0.07	1.90	0.01
Volatile material	75.16	0.13	74.14	0.16
Fixed carbon	23.05	0.36	18.93	0.08

* Statistically significant difference (*p* ≤ 0.05) between methods. SD: standard deviation.

## Data Availability

All data supporting the reported results are available on request from the corresponding author.
